# Antifungal Compound Isolated from* Catharanthus roseus* L. (Pink) for Biological Control of Root Rot Rubber Diseases

**DOI:** 10.1155/2018/8150610

**Published:** 2018-03-05

**Authors:** R. Zahari, N. Halimoon, M. F. Ahmad, S. K. Ling

**Affiliations:** ^1^Laboratory of Sustainable Bioresource Management (BIOREM), Institute of Tropical Forestry and Forest Products (INTROP), Universiti Putra Malaysia, Serdang, Selangor, Malaysia; ^2^Department of Environmental Science, Faculty of Environmental Studies, Universiti Putra Malaysia, 43400 Serdang, Selangor, Malaysia; ^3^Department of Biodiversity, Forest Research Institute Malaysia (FRIM), Kepong, Selangor, Malaysia; ^4^Department of Natural Product, Forest Research Institute Malaysia (FRIM), Kepong, Selangor, Malaysia

## Abstract

*Rigidoporus microporus, Ganoderma philippii, *and* Phellinus noxius *are root rot rubber diseases and these fungi should be kept under control with environmentally safe compounds from the plant sources. Thus, an antifungal compound isolated from* Catharanthus roseus *was screened for its effectiveness in controlling the growth of these fungi. The antifungal compound isolated from* C. roseus *extract was determined through thin layer chromatography (TLC) and nuclear magnetic resonance (NMR) analysis. Each* C. roseus* of the DCM extracts was marked as CRD1, CRD2, CRD3, CRD4, CRD5, CRD6, and CRD7, respectively. TLC results showed that all of the* C. roseus* extracts peaked with red colour at Rf = 0.61 at 366 nm wavelength, except for CRD7. The CRD4 extract was found to be the most effective against* R. microporus* and* G. philippii *with inhibition zones of 3.5 and 1.9 mm, respectively, compared to that of other extracts. These extracts, however, were not effective against* P. noxius.* The CRD4 extract contained ursolic acid that was detected by NMR analysis and the compound could be developed as a biocontrol agent for controlling* R. microporus* and* G. philippii.* Moreover, little or no research has been done to study the effectiveness of* C. roseus* in controlling these fungi.

## 1. Introduction

There are high demands for* Hevea brasiliensis* (rubber) tree throughout the world, especially in the rubber and wood fiber industries, and these contribute to a country's economy, for example, in Malaysia [1]. For many years, rubber trees have been attacked by root rot diseases caused by* Phellinus noxius, Rigidoporus microporus, *and* Ganoderma philippii *which are among the most common fungi in rubber plantations [2, 3]. According to [4] these fungi cause considerable damage to rubber plantations in Malaysia involving 18% of over 700-hectare land. The rubber species was also reported by [4] to suffer high mortality with more than 70000 trees over 2750 ha in estates due to* R. microporus *fungus. Currently, these diseases are kept under control using chemical fungicides [5]. However, these fungicides have posed various problems for the environment and ecosystems [6]. Therefore, an environmentally safer method to control these diseases is highly warranted. According to [7], some plant extracts contain environmentally safe compound and have the potential to be used as the much needed biopesticides to control plant diseases. One of the plant extracts containing various antifungal compounds is derivatives of* C. roseus*. Specifically, the plant contains 2,3-dihydroxybenzoic acid, 3,10-dinitrodiftalone, and desmethylomifensine, which have been shown to be effective against various fungi such as* Pythium aphanidermatum*,* Aspergillus fumigatus, Candida albicans, P. chrysogenum, *and* A. niger *[8, 9]. The callus from flower extracts of the plant was also effective against* A. niger, C. albicans, *and* Candida lipolytica* [10]. The leaves of* C. roseus *contain a phenolic such as 2,3-dihydroxybenzoic acid, an antifungal compound that was active against* P. aphanidermatum* [8]. Leaves of* C. roseus *in ethanol extracts also showed effective activity against* Fusarium moniliforme *with inhibition zone of 2.0 mm [11].

As reported by [12],* C. roseus* contains a large number of terpenoids and over 130 compounds have been isolated and identified [13]. The terpenoids can exist in lipophilic or hydrophilic, volatile or nonvolatile, cyclic or acyclic compounds [14]. The compound may have several chemical structures and possess antifungal properties. Diterpenes compound exhibits antifungal activity and is effective against* C. albicans *[15]. Terpenes are a group of carvone derivatives, and the compound also possesses antifungal activities [14]. In addition, several types of terpenoids or terpenes (C_10_H_16_), such as diterpenes (C_20_), triterpenes (C_30_), tetraterpenes (C_40_), hemiterpenes (C_5_), and sesquiterpenes (C_15_), have also effective antifungal activity [16]. Some of these compounds contribute to membrane disruption by lipophilic mode of action [14]. According to [16], diterpenoids increase hydrophilicity of kaurene extract; however, the methyl group has drastically reduced their antimicrobial activity.

Isolated* C. roseus* also contains ursolic acid (triterpene) and this compound is known to possess a wide range of more biological, anticancer [17], antimicrobial [18], hypoglycemic [19], antiprotozoal (against* Plasmodium falciparum*) [20], antiplatelet aggregation, anti HIV-ADIS, anti-mycobacterium tuberculosis, anti-inflammatory [18], and antioxidant [21] activities. Although* C. roseus* has various chemical compounds and biological activities, not much attention has been given to this particular plant, and there is little or no research done on its effectiveness in controlling* P. noxius, R. microporus, *and* G. philippii.* Thus, there is a need to carry out* in vitro* tests to determine antifungal activities of the compound isolated from* C. roseus* as a biocontrol agent against these fungal pathogens.

## 2. Materials and Methods

### 2.1. Preparation of Crude Extract and Thin Layer Chromatography (TLC)

Stems of* C. roseus *(flower is pink in colour) were collected from Terengganu, Malaysia.* C. roseus* was extracted with dichloromethane (DCM), as described by [23]. Column chromatography was used to separate chemical compounds present in the* C. roseus* extract. A total of 200 g of silica gel 60 F254 (Merck) with 5 cm of diameter and 24 cm of high in column chromatography was used. Silica gel was washed with hexane and placed in a column. About 20 g of* C. roseus *crude extract was dissolved in 100 mL of chloroform. The extract was mixed with 30 g of silica gel 60 F254 (Merck). Then, the mixtures of crude extracts were evaporated at 40°C to remove the chloroform. This procedure was known as a preabsorbing method. Then, the extracts were transferred to the bed of silica gel 60 F254 (Merck) with 60 g in the column. Firstly, 9 : 1 ratio of hexane and ethyl acetate was contained in the column to remove oil and chlorophyll pigments. The ratios of solvent used in the column are shown in Table 1. About 100 mL of samples from the column were contained in each test tube.

A droplet of each sample from a test tube was dropped on TLC plate [silica gel 60 F254, layer thickness: 0.28 mm (Merck, 10 × 20 cm)]. The plates were observed for pigment detection under UV absorbent at 366 nm. Then, the solutions from the test tube were separated for seven solutions based on their similarity in peaks that appeared on the TLC plates. The solutions are also known as extract fractions. These fractions were denoted as CRD to represent* C. roseus *dichloromethane and each extract fraction was marked as CRD1, CRD2, CRD3, CRD4, CRD5, CRD6, and CRD7. Among the extract fractions, CRD4 was transferred in the second column for peak separation.

### 2.2. Preparation of the CRD4 Extract and Nuclear Magnetic Resonance (NMR) Analysis

Sephadex was used in this study to get a single peak from the CRD4 extract. A total of 5 g of Sephadex LH20 (1.8 cm of diameter and 8 cm of high) was mixed with 100% methanol in a column chromatography. Then, chloromethane (CH_3_Cl) was added to the column. The CRD4 extract was diluted with chloromethane and added to the column. A Varian NMR system of 500 MHz spectrometer for ^1^H NMR and ^13^C NMR system was used in the study. Chemical shift was given in *δ* (ppm), and coupling constants were reported in Hz. The extract also was diluted with pyridine (C_5_D_5_N). A sample of 25 *μ*L was introduced via infusion using on-board syringe pump at a flow injection rate of 60 min.

### 2.3. Antifungal Activity

Each extract fraction was concentrated with DCM at 20 mg/mL. The* C. roseus *extract fractions were tested for antifungal activities as described by [23]. Three species of fungi (*R. microporus, G. philippii,* and* P. noxius*) were used for this study. DCM solvent without the plant extract was used as a control. After six days of incubation, the cultures were determined for the presence or absence of inhibition zone of fungi growing on PDA. The diameter (mm) of clear zone of inhibition was measured by using a calliper.

### 2.4. Statistical Analysis

A completely randomised design (CRD) was used in this experimental study for detection of antifungal activity. The experiment comprised a 7 × 3 factorial experiment with five replications. There were eight extract fractions separated (CRD1, CRD2, CRD3, CRD4, CRD5, CRD6, and CRD7) and three pathogens involved (*R. microporus, G. philippii,* and* P. noxius*). The inhibition zone (mm) of the fungal growth was analysed with analysis of variance (ANOVA) using SPSS software to compare the factors. Meanwhile, Tukey's range tests were used to compare the treatment means. If the count value (*p* value) of the inhibition zone was lower than 0.05, the effects of the treatments were assumed to be significant.

## 3. Results and Discussion

**Figure 1:**
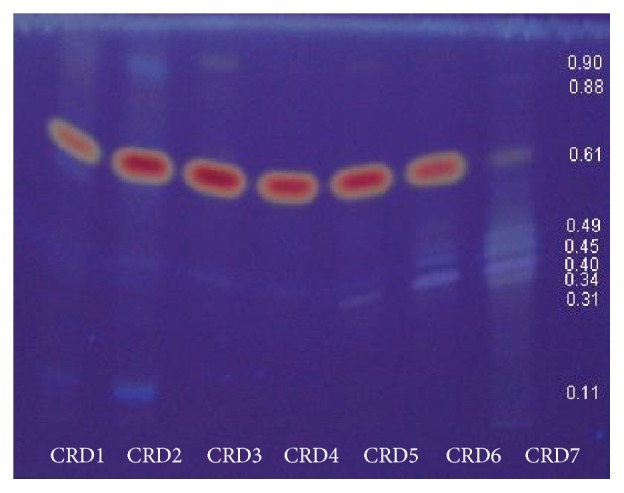
Peaks of* Catharanthus roseus* extract fractions from the TLC analysis after being sprayed with sulphuric acid at 366 nm.

**Figure 2:**
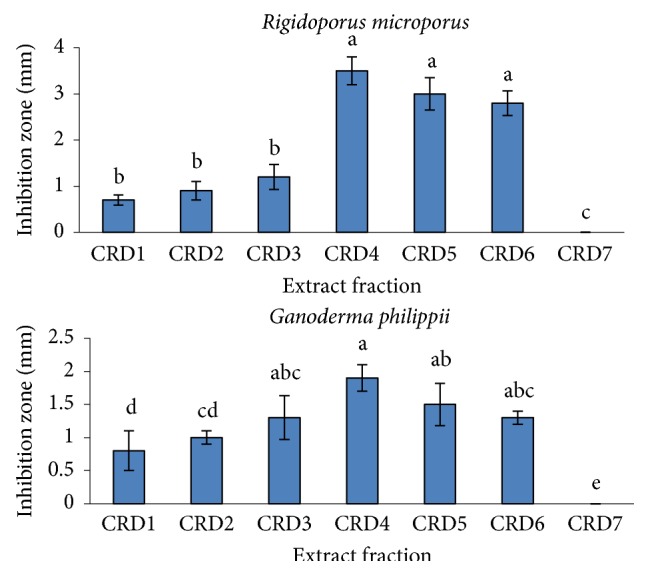
Mean inhibition zone in millimetre of* Catharanthus roseus* extract fractions obtained using dichloromethane against* Rigidoporus microporus *and* Ganoderma philippii*. Mean of each figure followed by the same letter is not significantly different based on Tukey HSD test (*p* ≤ 0.05).

**Figure 3:**
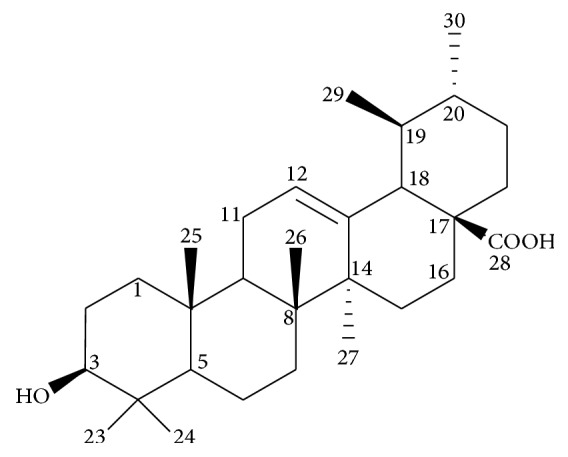
*3β-Hydroxyurs-12-en-28-oic acid* (ursolic acid).

A total of seven crude DCM extract fractions (CRD1, CRD2, CRD3, CRD4, CRD5, CRD6, and CRD7) isolated from* C. roseus* were obtained. Major peak at Rf = 0.61 was found to present in all the extract fractions under normal 366 nm wavelength after being sprayed with sulphuric acid, except for CRD7 fraction (Figure 1). However, CRD7 fraction produced colourful peaks at Rf = 0.34, 0.40, 0.45, and 0.49, respectively. The antifungal activity results of CRD1, CRD2, CRD3, CRD4, CRD5, CRD6, and CRD7 extract fractions showed that the antifungal effects were significant (*p* ≤ 0.05). The CRD4 extract fraction showed the maximum antifungal activity against* R. microporus* with an inhibition zone of 3.5 mm and* G. philippii* (1.9 mm) compared to that of other fractions (Figure 2). However, all the extract fractions were not effective against the growth of* P. noxius. *After chromatographic procedures, antifungal compounds were obtained from the CRD4 extract fractions. Analysis of their NMR system of 500 MHz for ^1^H NMR and ^13^C NMR system and comparison with published data (Table 2) allowed us to identify compounds as ursolic acid (Figure 3).

To confirm the major peak of* C. roseus *extract fraction (CRD4) at Rf = 0.61, a single compound was isolated from the extract and analysed by NMR. In this study, the single compound of CRD4 extract is also known as ursolic acid detected by an NMR analysis. Ursolic acid belongs to the triterpene group based on carbon number, that is, 30 carbons. This compound is known as 3*β*-hydroxyurs-12-en-28-oic acid. The result showed that ^1^H NMR gives two sets of doublet (*δ*0.98, *J* = 6.0 Hz, H-30; *δ*1.00, *J* = 7.5 Hz, 3H, H-29) of two secondary methyl groups, five tertiary methyl groups (*δ*1.21, H-23; 1.23, H-27; 1.04, H-25; 1.01, H-26; 0.87, H-24), and a trisubstituted olefinic double bond (*δ*5.52,* brs*) for H-12. The presence of an oxymethine proton resonating at *δ*3.44 (*brt*, *J* = 5.0, 9.5 Hz) for H-3 was revealed in the 1H-NMR of the ursolic acid (Figure 3).

**Figure 4:**
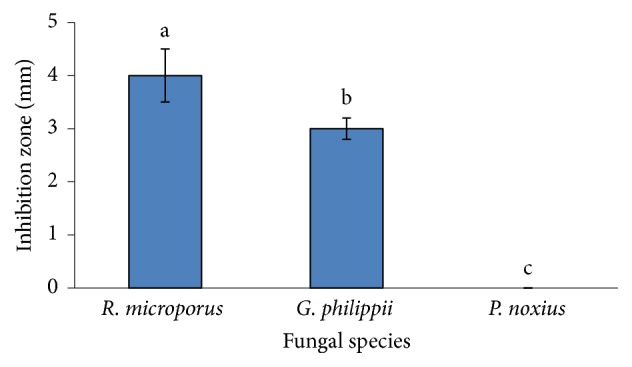
Mean inhibition zone in millimetre of ursolic acid from* Catharanthus roseus* extract fraction obtained using dichloromethane against* Rigidoporus microporus, Ganoderma philippii,* and* Phellinus noxius.* Mean of each figure followed by the same letter is not significantly different based on Tukey HSD test (*p* ≤ 0.05).

Ursolic acid from the CRD4 extract showed active antifungal activity against* R. microporus *and* G. philippii*. The extract fraction, however, showed no antifungal activity against* P. noxius *(Figure 4). The ursolic acid also acts as antimicrobial [18]. In relation to the structure and activity relationship, pentacyclic triterpenes possess a carboxylic acid functional group at carbon 17, which exhibits potent cytotoxicity [24]. This cytotoxicity was further demonstrated by [22] and they added that the compound might actively act as an antifungal. However, the toxics in* C. roseus *extract should be investigated in terms of its effect on human and environment. The ursolic acid also contains pentacyclic triterpene and has also acted as antimicrobial [18].

The major peaks might be contributed by triterpene. The red peak was also recorded from a previous study by [25], which indicated the presence of triterpene due to the sprayed sulphuric acid. In this study, the ratio of chloroform and methanol at 8 : 2 (v/v) was used and the solvent ratio produced major peaks in the* C. roseus* extracts. Depending on the complexity of the extracts, reducing pH of the mobile phase may reduce band broadening of the glycosides. It may also be caused by ionisation of the carboxyl groups not completely suppressed, while lowering the polarity may improve the acids close to the mobile phase front [26]. The weak absorbance may detect the existence of triterpene during the evaluation of the extracts. Similarly, the problem of absorbance also occurred during the determination of compounds in* Centella *extracts, in which the extracts generally have many colour reagents of triterpenoids, sapogenins, and saponins [26]. However, TLC cannot resolve isomers that may occur in trace quantities because a small number of theoretical plates are inherently associated with the technique.

The configuration of 3*β*-secondary hydroxyl group is in agreement with the observed coupling constant for 3*β*-hydroxyurs-12-en-28-oic acid [22]. The occurrence of doublet at *δ*2.63 (*J* = 11.5 Hz) for H-18, as well as a characteristic doublet of triplets signal (*δ*2.19, *J* = 4.4, *J*_2_ = 8.6, and *J*_3_ = 21.9 Hz, 1H) for H-16, suggests that the compound is an ursolic acid (Figure 3). In addition, Table 2 shows that chemical shifts of the carbon and proton were identical to those reported for 3*β*-hydroxyurs-12-en-28-oic acid. The compound was identified as ursolic acid by ^1^H and ^13^C from NMR data, as reported by [22]. In addition, the CRD4 extract fraction (at Rf = 0.61) was effective against* R. microporus *and* G. philippii *with 4.0 mm and 3.0 mm of inhibition zone, respectively, except for* P. noxius* (Figure 4). The results of the extract also showed that antifungal effects were significant (*p* ≤ 0.05).

For this reason, it is possible that the peak is triterpene. According to [27], triterpene present in* A. forbesii *extract has active antifungal activity against* Phytophthora botryosa*,* Phytophthora palmivora*, and* R. microporus*. The compound apparently appeared in the cell membranes of the plant species. These substances may have detergent properties and disrupt the cell membranes by invading fungal pathogens. As reported by [28], triterpene or triterpenoid may prove that the fungi can be eliminated by glycoalkaloid barrier in the plant cells. Triterpene or triterpenoid also consists of saponins bearing one or more sugar chains [28]. The saponin a-tomatine is a steroidal glycoalkaloid found in the root, stem, leaf, flower, and green fruit of tomato, comprising an aglycone moiety as tomatidine and also a tetrasaccharide moiety. The compound is composed of two molecules of glucose and one galactose and xylose [28]. The tomatine in saponins also has antifungal activities, and other saponins are attributed to their interaction with 3p-hydroxy sterols, causing an increase in membrane permeability, pore formation, and leakage of cells contents [29]. In addition, Steel and Drysdale [29] reported that tomatinase from* Botrytis cinerea *removes all four sugars by cleaving the *β*,l-linked galactose and releasing the tetrasaccharide lycotetraose and tomatidine [30]. However, most of the deglycosylation may be sufficient to destroy the ability of the E-tomatine to combine with membrane sterols of the plant and therefore eliminate its toxic effect on the fungi [30]. Thus, the detoxification of tomatine may prove that the fungi can be eliminated by glycoalkaloid barrier in the plant cells.

Apart from that, the leaf of* C. roseus *extract contains terpenoid indole alkaloids: chlorogenic acid, loganic acid, secologanin, vindoline, and oleanolic acid after being detected by NMR analysis [31, 32]. The chlorogenic acid has antifungal activity against* Aspergillus fumigatus *and* C. albicans*. Oleanolic acid and loganic acid from* Gentiana tibetica* also inhibit the growth of the pathogenic fungi* A. flavus *and* C. albicans *[33]. According to [34],* Breonadia salicina *extracted with DCM contains ursolic acid after being detected by NMR analysis.

## 4. Conclusion

In conclusion, the* C. roseus *extract isolated using TLC analysis produced several colours of the peaks. One major peak that appeared in CRD4 extract fraction at Rf = 0.61 showed the highest antifungal activity against* R. microporus *and* G. philippii *compared to the CRD1, CRD2, CRD3, CRD5, CRD6, and CRD7 extracts. These compounds, however, were not effective in controlling* P. noxius. *The single peak from CRD4 extract is known as ursolic acid after being detected by NMR analysis. In addition, the CRD4 extract has effective biological activities, especially against the growth of* R. microporus* and* G. philippii, *except for* P. noxius. *

## Figures and Tables

**Table 1:** Ratio of solvent in column chromatography.

Solvent	Ratio
Hexane and ethyl acetate	9 : 1
Hexane and ethyl acetate	8 : 2
Hexane and ethyl acetate	7 : 3
Hexane and ethyl acetate	6 : 4
Hexane and ethyl acetate	5 : 5
Hexane and ethyl acetate	4 : 6
Hexane and ethyl acetate	3 : 7
Hexane and ethyl acetate	2 : 8
Hexane and ethyl acetate	1 : 9
Chloroform and methanol	9 : 1
Chloroform and methanol	8 : 2

**Table 2:** Comparison of carbon in NMR chemical shift values of ursolic acid by [22] and *C. roseus* extract diluted with pyridine-*d*_5_ solution from the study.

Position	^13^C^*∗*^	^13^C	^1^H (*J*, H)^*∗*^	^1^H (*J*, H)
1	39.9	38.2	-	0.95
2	28.6	27.1	-	1.30
			-	1.80
			-	1.80
3	78.6	77.4	3.49 *dd*	3.44 (1H, *dd*, *J* = 5, 9.5 Hz)
4	39.5	37.9	-	-
5	56.3	54.8	-	1.10
6	19.3	19.6	-	1.50
7	34.1	35.5	-	1.60
8	40.0	38.2	-	-
9	48.5	47.3	-	1.55
10	37.9	37.6	-	-
11	24.1	23.1	-	1.90
12	126.1	124.9	5.52 *s*	5.47 (1H, *brs*)
13	139.8	138.5	-	-
14	43.0	40.8	-	-
15	29.2	29.3	-	1.15
			-	2.30
16	25.4	26.9	-	*δ* 2.19 (*d, J*_1_ = 4.4, *J*_2_ = 8.6, *J*_3_ = 21.9)
17	48.5	46.3	-	-
18	54.0	54.0	2.68 *d*	2.63 (1H, *J* = 11.5)
19	40.4	40.8	-	1.25
20	39.9	37.7	-	1.00
21	31.5	31.8	-	1.50
22	37.8	37.7	-	1.75
23	29.3	29.3	1.27 *s*	1.21 (3H, *s*)
24	17.1	17.0	0.91 *s*	0.97 (3H, *s*)
25	16.2	15.7	1.08 *s*	1.04 (3H, *s*)
26	18.0	17.0	1.05 *s*	1.01 (3H, *s*)
27	24.4	23.1	1.25 *s*	1.23 (3H, *s*)
28	180.4	178.1	-	-
29	17.6	17.0	1.03 *d*	1.00 (3H, *J* = 7.5 Hz)
30	21.9	21.8	0.98 *d*	0.98 (3H, *J* = 6.0 Hz)

^*∗*13^C NMR [500 MHz in C_5_D_5_N] [22]; ^*∗*1^H NMR [500 MHz in C_5_D_5_N] [22].
